# Distinct fecal microbial signatures are linked to sex and chronic immune activation in pediatric HIV infection

**DOI:** 10.3389/fimmu.2023.1244473

**Published:** 2023-08-29

**Authors:** Cecilia Rosel-Pech, Sandra Pinto-Cardoso, Monserrat Chávez-Torres, Nadia Montufar, Iván Osuna-Padilla, Santiago Ávila-Ríos, Gustavo Reyes-Terán, Charmina Aguirre-Alvarado, Norma Angelica Matías Juan, Héctor Pérez-Lorenzana, José Guillermo Vázquez-Rosales, Vilma Carolina Bekker-Méndez

**Affiliations:** ^1^ Posgrado en Ciencias Biológicas, Unidad de Posgrado, Universidad Nacional Autónoma de México (UNAM), Ciudad de México, Mexico; ^2^ Unidad de Investigación Médica en Inmunología e Infectología, Hospital de Infectología “Dr. Daniel Méndez Hernández”, Centro Médico Nacional “La Raza”, Instituto Mexicano del Seguro Social (IMSS), Ciudad de México, Mexico; ^3^ Centro de Investigación en Enfermedades Infecciosas, Instituto Nacional de Enfermedades Respiratorias Ismael Cosío Villegas, Ciudad de México, Mexico; ^4^ Hospital de Infectología “Dr. Daniel Méndez Hernández”, Centro Médico Nacional “La Raza”, Instituto Mexicano del Seguro Social (IMSS), Ciudad de México, Mexico; ^5^ UMAE Hospital General Dr. Gaudencio González Garza, Centro Médico Nacional La Raza, Instituto Mexicano del Seguro Social (IMSS), Ciudad de México, Mexico; ^6^ Hospital de Pediatría “Doctor Silvestre Frenk Freund”, Centro Médico Nacional Siglo XXI, Instituto Mexicano del Seguro Social, México, Mexico

**Keywords:** pediatric HIV infection, mother-to-child-transmission, gut microbiome, immune activation, inflammation, antibiotics, healthy eating index

## Abstract

**Introduction:**

Our understanding of HIV-associated gut microbial dysbiosis in children perinatally-infected with HIV (CLWH) lags behind that of adults living with HIV. Childhood represents a critical window for the gut microbiota. Any disturbances, including prolonged exposure to HIV, antiretroviral drugs, and antibiotics are likely to have a significant impact on long-term health, resulting in a less resilient gut microbiome. The objective of our study was to characterize the gut microbiota in CLWH, and compare it with HIV-unexposed and -uninfected children.

**Methods:**

We enrolled 31 children aged 3 to 15 years; 15 were CLWH and 16 were HUU. We assessed dietary patterns and quality; quantified soluble and cellular markers of HIV disease progression by flow cytometry, enzyme-linked immunosorbent and multiplex-bead assays, and profiled the gut microbiota by 16S rRNA sequencing. We explored relationships between the gut microbiota, antibiotic exposure, dietary habits, soluble and cellular markers and host metadata.

**Results:**

Children had a Western-type diet, their median health eating index score was 67.06 (interquartile range 58.76-74.66). We found no discernable impact of HIV on the gut microbiota. Alpha diversity metrics did not differ between CLWH and HUU. Sex impacted the gut microbiota (R-squared= 0.052, PERMANOVA p=0.024). Male children had higher microbial richness compared with female children. Two taxa were found to discriminate female from male children independently from HIV status: Firmicutes for males, and *Bacteroides* for females. Markers of HIV disease progression were comparable between CLWH and HUU, except for the frequency of exhausted CD4+ T cells (PD-1+) which was increased in CLWH (p=0.0024 after adjusting for confounders). Both the frequency of exhausted CD4+ and activated CD4+ T cells (CD38+ HLADR+) correlated positively with the relative abundance of Proteobacteria (rho=0.568. false discovery rate (FDR)-adjusted p= 0.029, and rho=0.62, FDR-adjusted p=0.0126, respectively).

**Conclusion:**

The gut microbiota of CLWH appears similar to that of HUU, and most markers of HIV disease progression are normalized with long-term ART, suggesting a beneficial effect of the latter on the gut microbial ecology. The relationship between exhausted and activated CD4+ T cells and Proteobacteria suggests a connection between the gut microbiome, and premature aging in CLWH.

## 1 Introduction

In July 2022, the World Health Organization (WHO) reported the estimated global human immunodeficiency virus (HIV) prevalence across all ages; there are 38.4 million (M) people living with HIV (PLWH) (1). Of those, 1.7M are children aged 15 and less. In 2021, there was a 32% and 52% global decrease in new infections and HIV-related deaths relative to 2010, respectively. Still, 650,000 PLWH died of HIV-related complications, of these 98,000 were children. This decline in HIV-related deaths over time is largely due to the increased availability and effectiveness of antiretroviral therapy (ART) and continuous efforts towards reaching the 95-95-95 targets of the HIV service cascade. Globally, 75% (28.7M) of PWLH received ART in 2021. In Mexico, the estimated number of PLWH is 227,378. The cumulative number of perinatally-infected children aged 0 to 14 years is 4,229 (2). In 2023, 14 children were perinatally-infected with HIV [CLWH, (2)]. The estimated ART coverage among adults and children is 61% and 49% respectively, 54% are virally-suppressed, and the mother-to child transmission is 0.3%. Although the gap in life expectancy is gradually decreasing, PLWH (adults and children alike) are still at a higher risk of noncommunicable diseases (NCD) compared to the general population (3). This is partly due to persistent immune dysfunction and chronic inflammation which fail to normalize despite effective suppressive ART (4, 5).

In 2006, a seminal paper published in Nature Medicine concluded that microbial translocation, the passage of microbial products and/or bacteria from the gut to the systemic circulation, is a key driver of chronic inflammation and HIV pathogenesis, linking the gut microbiome to HIV disease progression (6, 7) and NCD (5, 8–10). This study (and others) conceptualized the current paradigm that functional (massive depletion of gut T helper 17 cells) and structural (loss of tight junctions) events secondary to HIV infection cause lasting damage to the gut mucosa, providing favorable conditions for alterations in the composition and function of gut microbial communities to occur (5, 11–14). These alterations are commonly referred to as microbial dysbiosis. Collectively, studies in adults PLWH (ALWH) have shown a decrease in bacterial (12) and fungal diversity (15, 16), an expansion of the virome (9, 17), an increase in pathobionts and a decrease in anti-inflammatory commensals (14), compared with seronegative individuals. Microbial dysbiosis is partially restored by ART. Also, studies have shown that ART is linked to a distinct microbial dysbiosis independent from HIV (18–21). Less is known in CLWH, as only a handful of studies have been published (22–24). Contrary to ALWH, whose studies have been published in high income countries (for the most part), studies in CLWH have been conducted in Africa [Cameron (24), Zimbabwe (25), South Africa (26)], India (22), and Italy (27). Collectively, studies suggest similarities between ALWH and CLWH, despite differences in mode of HIV transmission (vertical versus horizontal), absence of some confounding variables (sexual preference) and the presence of confounders specific to CLWH [use of co-trimoxazole, a prophylactic antibiotic recommended by the WHO in countries with high burden of malaria and bacterial infections (28)].

Understanding of HIV-associated gut microbial dysbiosis in CLWH lags behind that of ALWH. Since childhood represents a critical window for the gut microbiota (development and maturation), and the immune system (education), disturbances are likely to have a significant impact on long-term health, quality of life and longevity (29–31). Also, prolonged exposure to HIV, ART, antibiotics (32) and maternal factors (33), may result in a less resilient gut microbiome and a compromised gut microbiome-immune system axis, increasing tolerance to pathobionts. This, in turn, may lead to increased prevalence of cardiometabolic alterations, and other NCD (and their premature clinical presentation compared to the general population). The objective of our study was to characterize the gut microbiota in CLWH in Mexico, and compare it with HIV-unexposed and -uninfected children (HUU). We used dietary questionnaires and healthy eating index (HEI) scores to assess dietary patterns and quality; flow cytometry to quantify CD4+ and CD8+ T cell immune activation, senescence, exhaustion and cell cycling; enzyme-linked immunosorbent assays to quantify markers of inflammation, bacterial and fungal translocation, enterocyte integrity, and adipose tissue inflammation, multiplex-bead assay to quantify soluble markers of immune activation, and profiled the gut microbiota using 16S rRNA sequencing using fecal samples. We also explored relationships between the gut microbiota, antibiotic exposure, dietary habits, soluble and cellular markers of HIV disease progression and host metadata.

## 2 Methods

### 2.1 Ethics statement

This study was conducted according to the Declaration of Helsinki and its later amendments. Ethical approval for this study was obtained from the Instituto Nacional de Enfermedades Respiratorias (INER, approval number C23-20) and the Instituto Mexicano del Seguro Social (IMSS, approval number R-2018-785-130). Written informed consent and age-appropriate assent were obtained from all participants and/or parents before study enrollment.

### 2.2 Study participants

We enrolled a total 31 children aged 3–15 years between 2019 and 2020; 15 were perinatally infected with HIV; here referred to as children living with HIV (CLWH) and 16 were HIV-unexposed and uninfected children (HUU). All children were enrolled from three pediatric services within IMSS. CLWH were enrolled from Servicios de Infectología Pediátrica, Centro Médico Nacional (CMN) La Raza and Servicios de Infectología Pediátrica, CMN, Siglo XXI. HUU were enrolled from Servicio de Cirugía Pediátrica, Hospital General, CMN “La Raza”.

### 2.3 Research samples PBMC, plasma and stools

Whole blood (8mL) was collected by a phlebotomist by venipuncture in Vacutainer® EDTA tubes (ethylenediaminetetraacetic acid, BD, New Jersey, United States). Blood samples were immediately processed to separate plasma by centrifugation (3,000 rpm for 10 min) and cryopreserved at −80°C. Next, peripheral blood mononuclear cells (PBMC) were isolated by Ficoll density gradient centrifugation (STEMCELL technologies, Vancouver, Canada) and stored at -196°C. Stool samples were collected by each participant and processed immediately. First, stools were classified according to the Bristol Stool Chart (34), then 220 mgs of feces were aliquoted in 2mL Eppendorf LoBind tubes, and stored at -80°C.

### 2.4 Nutritional assessment

Anthropometric and dietary assessment were performed by qualified dietitians. First, height and weight were measured using a Seca mechanical scale with a stadiometer (Seca, Connecticut, United States*)*. Body mass index (BMI) and BMI-for-age (BMI/A) were calculated in accordance to the WHO´s reference values using the WHO AnthroPlus (35). Dietary assessment was conducted using semi-quantitative methods including a food frequency questionnaire (FFQ) and three 24-hour dietary recalls validated for the Mexican population as previously described (36). Estimated nutrient and energy intake were determined using the NutriSurvey software (37) and total energy consumed was expressed in kilocalories (kcal), macronutrients, and micronutrients in grams (g) and milligrams (mg), respectively. Diet quality was assessed using the 2010 Healthy Eating Index [HEI, (38)] and information derived from three 24-hour dietary recalls. HEI is a score system that recapitulates how well individuals adhere to dietary recommendations for Americans. This score system includes two types of components distributed across 12 groups (total fruit, whole fruit, total vegetables, greens and beans, whole grains, dairy, total protein foods, seafood, plant proteins, fatty acids, refined grains, sodium, and empty calories) and ranges from 0 to 100, 100 denoting excellent compliance to dietary recommendations. The HEI score was further categorized into healthy (HEI score above 80), needs changes (HEI score between 50-79) and unhealthy (HEI score below 50).

### 2.5 Flow cytometry

Canonical markers of immune activation (CD38 and HLADR), senescence (CD57), exhaustion (programmed cell death 1, PD-1) and cell cycling (Ki67) were quantified in both CD4+ and CD8+ T lymphocytes using flow cytometry as previously published (39) with minor modifications. Monoclonal antibodies used in this study are listed in **Supplementary Table 1**. The LIVE/Dead™ fixable blue dead stain was used to exclude dead cells (viability marker, ThermoFisher Scientific). A dump gate was added to exclude unwanted populations. Briefly, PBMCs were thawed and rested for at least 30 min. Cells were washed in 15mL of phosphate buffered solution (PBS), centrifuged at 500 x *g* for 5 min at room temperature (RT). After discarding the supernatant, cells were stained with a cocktail of monoclonal extracellular antibodies for 20 min in the dark. Cells were washed twice in PBS and permeabilized with Fix/Perm kit (ThermoFisher Scientific) for intracellular staining (Ki-67) and incubated for 60 min. Cells were washed twice in PBS, and fixed in 300µL of 1% paraformaldehyde (Sigma-Aldrich, St. Louis, Missouri, USA) and kept in the dark at 4°C until acquisition. Flow cytometry acquisition was performed on a BD LSRFortessa (BD Biosciences, San Jose, CA, USA) within 24 h of staining. A minimum of 3 million events were acquired per sample. Quality controls were performed using BD cytometer Setup and Tracking Beads and rainbow beads (BD biosciences). A compensation matrix was calculated and applied using BD Comp Beads (BD Biosciences). Data was analyzed using FlowJo™ v10.9 (BD, Ashland, Oregon, Unites States). Raw FCS (flow cytometry standard) files were first quality-controlled using FlowAI v2.3.1 (40) with default parameters, except for the changepoint penalty which was set to 500. A mean of 2,529,571 events were retained after quality-control. Populations were pre-gated for singlets and morphology by using forward versus side scatter, followed by the exclusion of dead cells (leaving only aqua dye negative events). Live cells were gated on CD3+ and then on CD4+ or CD8+ T-cells. A representative gating strategy is shown in **Supplementary Figure 1**. FMO (fluorescence minus one) were used to accurately set the gates for positive populations.

### 2.6 Quantification of markers by ELISA

Markers of microbial and fungal translocation (soluble CD14, sCD14, 1,3-β-D-Glucan), epithelial barrier integrity (intestinal fatty binding acid protein, I-FABP), inflammation (C reactive protein, CRP), and adipose tissue inflammation (sCD163, monocyte chemoattractant protein-1, MCP-1 and adiponectin) were measured in plasma using commercial enzyme-linked immunosorbent assays (ELISA) according to their respective package inserts. Human Quantikine Immunoassays were used for sCD14, sCD163, CRP, MCP-1, adiponectin (R&D systems, Minneapolis, Minnesota, USA), Hycult Biotech (Uden, The Netherlands) for I-FABP and MyBioSource (San Diego, CA, USA) for 1,3-β-D-Glucan. Plasma samples were diluted 250-fold for sCD14, 25-fold for sCD163, 3-fold for I-FABP and MCP-1, and 150-fold for CRP and adiponectin. For 1,3-β-D-Glucan, plasma was used neat. Assays were read at 450nm (Epoch, BioTek, Winooski, Vermont, USA) and analyzed using the Gen5 software, version 2.07. Wavelength correction was applied by resting the optical density at 570nm. Samples were run in duplicate for all assays.

### 2.7 Quantification of cytokines by multiplex-bead assay

Plasma samples were thawed, mixed and clarified by centrifugation (14,000 x *g* for 10 min at 4°C). The Human Ultrasensitive Cytokine Magnetic 10-Plex panel (ThermoFisher Scientific) was used to quantify granulocyte-macrophage colony-stimulating factor (GM-CSF), interferon [IFN]-γ, interleukin [IL]-1β, IL-2, IL-4, IL-5, IL-6, IL-8, IL-10, and tumor necrosis factor [TNF]-α) in plasma. This 10-Plex cytokine assay was run according to the manufacturer´s instructions. All samples were run in duplicate and read with the Luminex™ 200™. Cytokine concentrations were determined from each standard curve using a 5-parameter logistic weighted algorithm (Xponent ™ v3.1 software, Luminex™, Austin, Texas, USA). Cytokines with at least 75% of values within the inferior and superior limit of detection set by each respective standard curve were used for subsequent analyses. These included IL-2, IL-4, IL-5, IL-6, IFN-γ, and TNF-α.

### 2.8 16S rRNA gene sequencing

DNA was extracted using the PowerSoil DNA Extraction Kit (MO BIO Laboratories, Carlsbad, CA, USA) and libraries were prepared as described in the MiSeq 16S rRNA gene Amplicon Sequencing protocol. Primers for the V3–V4 region primers (41) were: forward (341F): 5′-CCTACGGGNGGCWGCAG-3′; and reverse (805R): 5′- GACTACHVGGGTATCTAATCC-3′. Primers were purchased with overhang adapter sequences, the forward overhang sequence was: 5′- TCGTCGGCAGCGTCAGATGTGTATAAGAGACAG-followed by the 341F primer; and the reverse overhang sequence was 5′-GTCTCGTGGGCTCGGAGATGTGTATAAG AGACAG-followed by the 805R primer (Invitrogen, Waltham, MA, USA). Briefly, each DNA sample was amplified in triplicate by PCR in a final volume of 25μL. The PCR mix included the V3–V4 overhang adapter sequences (1μM), 5ng/μL of DNA, and a Platinum *Taq* DNA High Fidelity Polymerase (Invitrogen). PCR cycling was limited to 25 cycles. PCR amplification was verified on a 2% agarose gel (Sigma-Aldrich, MO, USA). Triplicate PCR reactions were pooled per sample and purified using AgenCourt AMPure XP beads (Beckman Dickson, Atlanta, GA, USA) as instructed by the manufacturer. Dual indices were attached by PCR to the purified PCR amplicon using the Nextera XT Index Kit (Illumina, San Diego, CA, USA). The index PCR clean-up step was repeated to guarantee complete purification of the indexed libraries using AgenCourt AMPure XP beads. Library quantification was performed using the Qubit dsDNA HS Buffer and Standards kit (Invitrogen) as instructed by the manufacturer. Next, each library was normalized to 4nM by dilution and pooled together. Libraries size and molarity were checked on a 2100 Bioanalyzer instrument (Agilent Technologies, Santa Clara, CA, USA) and sequenced on the Illumina MiSeq™ platform using a final library molarity of 14pM to obtain paired-end sequences (2×300 cycles). The internal PhiX control was used at 25% (also at 14pM). Negative controls were included in parallel to account for contamination from reagents during DNA extraction and library preparation. Control samples did not amplify by PCR nor did they yield a quantifiable library, verified on the 2100 Bioanalyzer. Library preparation, and sequencing was performed at the Centro de Investigación en Enfermedades Infecciosas, INER. Raw 16S sequences were deposited at the National Center for Biotechnology Information-Sequence Read Archive (NCBI-SRA) under project PRJNA868762 (https://www.ncbi.nlm.nih.gov/sra/PRJNA868762).

### 2.9 16S rRNA gene sequence analysis

Raw demultiplex FastQ files (read 1 and read 2) were processed using QIIME2 [Quantitative Insights into Microbial Ecology, version 2022.2, (42)]. First, sequences were imported into QIIME2, primers were removed using qiime cutadapt trim-paired, and checked for sequence quality and length. Next, DADA2 (43) was used to denoise, remove chimera and PhiX sequences, merge paired-end reads and construct an amplicon variant sequences (ASVs) table. Negative controls yielded less than 1000 raw sequences (read 1 and read2) and no paired-sequences were obtained after DADA2. One participant (CLWH) was excluded due to low number of sequences (<1000). After excluding this sample, a total of 4344355 paired-sequences were retained, with a median of 143,403.5 (min, 65,696-max, 238,800) per sample. Taxonomy was assigned using the SILVA database (44), release 138, at 99% identity, pre-trained for the V3-V4 16S rRNA region. A phylogenetic tree was constructed using qiime phylogeny align-to-tree-mafft-fasttree. Qiime2 artifacts were imported into R for further manipulation, and graph visualization (R version 4.3.0). Rarefaction was performed at a sampling depth of 59,126 sequences per sample. Alpha-diversity was estimated using Shannon index, observed ASVs (richness), and Faith phylogenetic distance, while beta-diversity was estimated using Bray-Curtis dissimilarity index (vegan v.2.6-4, R). We also explored other beta-diversity metrics (unweighted and weighted Unifrac) and found they compared well with Bray-Curtis, with no additional information gained from these two metrics (data not shown). Beta-diversity was visualized using principal coordinate analysis (PCoA). Permutational Multivariate Analysis of Variance (PERMANOVA, adonis2, vegan, with 10,000 permutations) was used to test differences between groups. Multi-factor models were also run with multiple explanatory variables (sex, HIV status and age) using the adonis2() function to test for the interaction between these 3 variables. Homogeneity of group dispersion was verified with the *betadisper* function (vegan, R). Linear discriminant analysis (LDA) effect size [LEfSe, (45)] was performed using default parameters and an LDA score equal to or above 4. To test for discriminant taxa by HIV status (and sex assigned at birth: female vs male, hereby referred to as sex only), LEfSe was performed on the unrarefied ASV table, filtered to remove ASV found in fewer than 10% of samples. This analysis was run on the Galaxy web application (https://huttenhower.sph.harvard.edu/galaxy/).

### 2.10 Statistical analysis

Data were expressed as number (percentage), median (interquartile range), mean (plus or minus standard deviation) or number (percentage) as appropriate. Groups were compared using the Wilcoxon Rank Sum Test for continuous variables and Fisher´s Exact test or Chi-square test (as required) for categorical values (GraphPad Prism v8, San Diego, California, United States). Correlation coefficients and p values were computed with the nonparametric Spearman test using “cor.test” function in R, p values were adjusted using the false discovery rate (FDR). Regression analyses were used to assess relationships between HIV status and numeric outcome variables using the “lm” function in R, and adjusted for sex, age, BMI/A (model 1) or sex, age, BMI/A and antibiotic use within the last 3 months (model 2). Given the impact of sex on the gut microbiota structure (as assessed by PERMANOVA), we also fitted regression analyses between numeric outcome variables of interest and sex using the “lm” function, adjusting for HIV status, age, BMI/A (model 1) and HIV status, age, BMI/A and antibiotic exposure within the last 3 months (model 2). Data were visualized using “ggplot2”, ggcorrplot2” and “ampvis2”.

## 3 Results

### 3.1 Cohort characteristics

A total of 31 children were enrolled; 15 were CLWH and 16 were HUU. Children were aged between 3 and 15 years old, with a mean of 9.53 years. **Table 1** summarizes the cohort characteristics. This cohort was well-balanced in terms of number of participants per group, sex, age, BMI/A, and immune status (as assessed by CD4+ T cell count). All CLWH were on antiretroviral therapy (ART) for a median of 8.65 years (interquartile range 7.57-10.75) and were virally suppressed (plasma viral load <200 copies/mL). Ten CLWH were on protease inhibitors (PI, 66.7%), 4 on integrase strand transfer inhibitors (InSTI, 26.7%) and 1 on non-nucleoside reverse transcriptase inhibitors (NNRTI, 6.7%). No CLWH had CD4+ T cell counts below 200 cells/µL. **Supplementary Table 2** summarizes the HIV clinical parameters of CLWH at HIV diagnosis and at study enrollment.

**Table 1:** Cohort characteristics.

	CLWH	HUU	P value
# of participants	15	16	
Sex at birth: Female	8 (53.3)	6 (37.5)	0.47
Age (years)	10.20 ± 2.54	9.06 ± 2.82	0.248
Residence Mexico City State of Mexico Others	6 (40.0) 6 (40.0) 3 (20.0)	3 (18.8) 12 (75.0) 1 (6.3)	0.25
BMI (kg/m^2^) BMI/A (z score)	17.485 ± 2.84 0.34 (-0.92-1.45)	18.954 ± 3.744 1.01 (0.12-1.75)	0.40 0.105
BMI/A category Underweight Normal Overweight Obesity	2 (13.3) 9 (60) 4 (26.7) 0 (0)	0 (0) 8 (50) 6 (37.5) 2 (12.5)	0.29
CD4+ T cell count (cells/µL) CD4/CD8 ratio	905 (718-1237) 0.86 (0.59-0.927)	928.5 (623-1454) 1.32 (1.050-1.722)	0.93 **0.004**
Mode of delivery Vaginal Caesarean section	9 (60.0) 6 (40.0)	5 (31.3) 11 (68.8)	0.15
Feeding Breastfed exclusively Formula exclusively Both	9 (60) 2 (13.3) 4 (26.7)	7 (43.8) 1 (6.2) 8 (50)	0.43
HEI score	66.35 (58.26-71.05)	69.41 (64.55-76.52)	0.20
HEI category Healthy Need changes Unhealthy	1 (6.67) 13 (86.67) 1 (6.67)	1 (6.25) 14 (87.5) 1 (6.25)	

Data are expressed as number (percentage), median (interquartile range), mean (± standard deviation) or number (percentage) as appropriate. Groups were compared using the Wilcoxon Rank Sum Test for continuous variables and Fisher´s Exact test or Chi-square test (as appropriate) for categorical values (GraphPad Prism v8). BMI/A classification was based on the World Health Organization. The healthy eating index was calculated as described in Methods.

BMI, body mass index; BMI/A, BMI-for-age; CLWH, Children living with HIV; HEI, healthy eating index; HUU, HIV-unexposed and -uninfected children; IQR, interquartile range; kg, kilograms; m, meters; n, number; %, percentage; #, number; µL, microliter; -, not applicable.

### 3.2 Dietary patterns and dietary quality

Overall, children in this study (CLWH and HUU alike) had a Western-type diet, characterized by excessive intake of refined carbohydrates, animal protein, sugar, saturated fats and inadequate fiber consumption (**Supplementary Tables 3, 4**). Ten children had overweight and 2 had obesity (according to their BMI/A). The overall median HEI score was 67.06 (interquartile range 58.76-74.66). Two children (1 CLWH and 1 HUU) had a healthy diet. All other children had poor diets or needed changes (**Table 1**). After adjusting for multiple comparisons, no differences in dietary pattern were seen between CLWH and HUU (**Supplementary Tables 3, 4**).

### 3.3 Immune senescence remains elevated in CLWH despite antiretroviral therapy

Next, we assessed markers linked to HIV disease progression and pathogenesis, and overall health. We quantified soluble and cellular markers in plasma and peripheral blood mononuclear cells respectively, to determine levels of microbial translocation (bacterial and fungal), gut permeability, immune activation (plus senescence, exhaustion and cell cycling) and inflammation. We also included markers of adipose tissue inflammation (sCD163, adiponectin and MCP-1). The median and interquartile range of each marker is shown in **Table 2**. When comparing CLWH and HUU, no differences were found, except for the frequency of exhausted CD4+ T cells (PD-1+), which was elevated in CLWH compared with HUU (p=0.0092). The association between HIV status and the frequency of exhausted CD4+ T cells (PD-1+) was further evaluated using regression analysis and remained significant after adjusting for sex, age, BMI/A, and recent antibiotic use (p=0.0024, **Supplementary Table 5**).

**Table 2:** Soluble and cellular markers linked to HIV disease progression and pathogenesis.

	CLWH	HUU	P value
Microbial translocation sCD14 (ng/mL)	2125.82 (1978.24-2448.68)	1867.31 (1759.45-2123.83)	*0.09*
Enterocyte integrity I-FABP (pg/mL)	854.5 (549.1-2028)	751.6 (609.3-1594)	0.59
Inflammation CRP (ng/mL)	1534 (679.6-3210)	1793 (1019-5104)	0.46
T-cell activation CD4+ CD38+ HLADR+ (%) CD4+ CD57+ (%) CD4+ PD-1+ (%) CD4+ Ki67+ (%) CD8+ CD38+ HLADR+ (%) CD8+ CD57+ (%) CD8+ PD-1+ (%) CD8+ Ki67+ (%)	0.84 (0.59-1.19) 0.59 (0.29-1.42) 1.71 (1.38-2.33) 1.85 (1.33-2.85) 1.50 (0.75-4.32) 13.95 (5.60-27.18) 0.76 (0.50-1.31) 1.41 (0.70-2.57)	0.69 (0.44-1.10) 0.83 (0.16-2-01) 1.10 (0.59-1.41) 1.97 (1.67-2.35) 1.25 (0.67-1.87) 7.10 (3.09-15.80) 0.41 (0.34-1.14) 1.17 (0.84-1.71)	0.39 0.94 **0.0094** 0.93 0.44 0.158 0.155 0.83
Fungal translocation 1,3-β-D-glucan (pg/mL)	200.4 (167.5-225.3)	199.1 (153.3-265.3)	0.89
Cytokines IL-2 (pg/mL) IL-4 (pg/mL) IL-5 (pg/mL) IL-6 (pg/mL) IFN-γ (pg/mL) TNF-α (pg/mL)	1.02 (0.56-5.67) 5.95 (3.09-27.98) 0.82 (0.16-5.33) 0.83 (0.46-3.35) 0.30 (0.11-0.84) 6.72 (4.17-17.19)	1.60 (0.49-4.33) 15.39 (7.08-32.52) 2.25 (0.60-5.86) 1.37 (0.55-2.72) 0.49 (0.19-1.11) 10.19 (5.00-17.61)	0.97 0.29 0.52 0.61 0.63 0.57
Adipose tissue inflammation Adiponectin (µg/mL) MCP-1 (pg/mL) sCD163 (ng/mL)	18.19 (10.78-23.41) 157.3 (146.5-208.2) 596.8 (489.8-696.0)	17.53 (13.06-21.60) 156.7 (113.1-205.6) 536.9 (414.8-666.9)	0.89 0.42 0.20

Data are expressed as median (interquartile range). Groups were compared using the Wilcoxon Rank Sum Test. Soluble markers were quantified in plasma by enzyme-linked immunosorbent assay and multiplex-bead assay, and cellular markers were quantified by flow cytometry using thawed peripheral blood mononuclear cells as described in methods.

%, percentage; CD, cluster of differentiation; CLWH, children living with HIV; CRP, C reactive protein; HUU, HIV-unexposed and -uninfected children; I-FABP, intestinal fatty acid binding protein; IL, interleukin; IFN-γ, interferon gamma; MCP-1, monocyte chemoattract protein-1; TNF-α, tumor necrosis factor alpha; s, soluble.

### 3.4 No discernable impact of HIV on the gut microbiota

**Figure 1:**
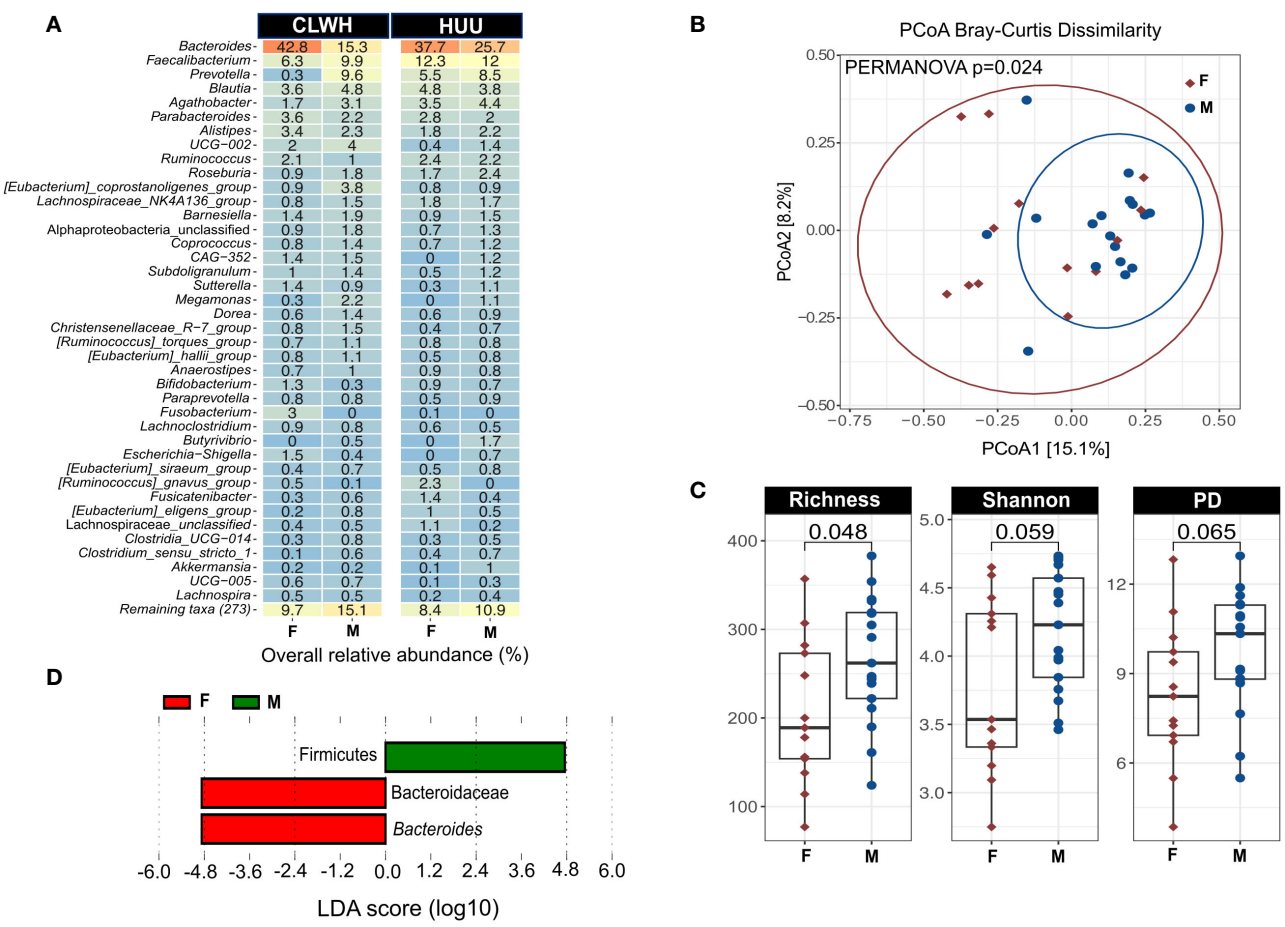
Sex impacts the gut microbiota of children independently of HIV status. **(A)** Heatmap showing the mean overall relative abundance of the top 40 genera stratified by HIV status and faceted by sex. **(B)** PCoA showing the impact of sex on the gut microbiota structure based on the Bray-Curtis dissimilarity index (PERMANOVA p=0.024). Sex explains 5.2% of the variation in bacterial community structure. Points are colored by sex (females in red, males in blue), ellipses represent 95% confidence interval for each group. **(C)** Alpha diversity metric comparisons between female and male children using Wilcoxon rank sum test, showing the male children have increased richness compared to female children. **(D)** Linear discriminant analysis (LDA) effect size (LEfSe), showing the two taxa that discriminate female and male children: Firmicutes for male children, *Bacteroides* for female children. Graphs were created using ampvis2 version 2.8, and ggplot2 version 3.4.2. %, percentage; CLWH, children living with HIV; F, female; HUU, HIV-unexposed and uninfected children; M, male; PCoA, principal coordinate analysis; Faith's phylogenetic diversity (PD) PERMANOVA, Permutational Multivariate Analysis of Variance.

Next, we profiled the gut microbiota using fecal samples and 16S rRNA sequencing. Alpha diversity was higher in CLWH for all three indices (richness, shannon and phylogenetic distance), although no differences were found when comparing with HUU (**Supplementary Figure 2A**). Regression analyses confirmed no clear association between HIV and alpha diversity (**Supplementary Table 6**). Next, bacterial community structure was visualized using principal coordinate analysis (PCoA, Bray-Curtis dissimilarity index). We found no clear evidence of clustering by HIV status (CLWH compared with HUU, R-squared= 0.034, PERMANOVA p=0.46, **Supplementary Figure 2B**). The Bray-Curtis dissimilarity index was similar between CLWH and HUU, showing a high degree of interindividual variation within groups (**Supplementary Figure 2C**). Next, we explored the taxonomic composition at phylum level (**Supplementary Figure 3**). Firmicutes and Bacteroidota were predominant with an overall mean relative abundance of 50.67% and 44.26%, respectively. These two phyla accounted for over 94% of all phyla present. At genus level, *Bacteroides* was predominant (29.69%), followed by *Faecalibacterium* (10.24%), *Prevotella* (6.23%), *Blautia* (4.18%), *Agathobacter* (3.27%), *Parabacteroides* (2.60%), *Alistipes* (2.39%), *UCG-002* (Oscillospiraceae, 1.94%), *Ruminococcus* (1.93%), and *Roseburia* (1.79%). **Figure 1A** summarizes the overall relative abundance of the top 40 genera; these account for 89.44% of all genera present. Heatmap showing the relative abundance of the top 40 genera in each child is shown in **Supplementary Figure 4**. To assess if there were discriminant ASVs between CLWH and HUU (i.e., by HIV status), we performed linear discriminant analysis (LDA) effect size (LEfSe, using default parameters, the threshold on the LDA was set to 4 or above). No discriminant taxa between CLWH and HUU were found using LEfSe.

### 3.5 Sex impacts the gut microbiota of children

Next, we explored the influence of other variables known to impact the gut microbiota, including sex, age, BMI/A, previous antibiotic use, ART, place of residence, immune status, maternal factors, siblings, and stool consistency. Information regarding previous antibiotic usage and stool consistency is summarized in **Supplementary Tables 6, 7**, respectively. To assess the impact of these variables on bacterial community membership, we used PERMANOVA (10,000 permutations). Results on the impact of each variable in single-variable models are shown in **Supplementary Table 8**. Sex was the only variable that significantly impacted the gut microbiota (R-squared= 0.052, PERMANOVA p=0.024, **Figure 1B**), though sex accounted for a relatively small amount of the variation (5.2%) and the PERMANOVA p was not adjusted for multiple comparisons. While HIV status and age did not significantly impact the gut microbiota in single-variable models, we also explored the interaction of sex, HIV status and age using adonis2 function. This multi-factor analysis yielded similar results to the single-variable model (R2 = 0.048, PERMANOVA p=0.044), suggesting that sex impacts the gut microbiota independently of age and HIV status. We also found that male children had higher alpha diversity (richness only) compared with female children (**Figure 1C**). Two ASV were found to discriminate female from male children using LEfSe (LDA>4): Firmicutes for males, and *Bacteroides* for females (**Figure 1D**). To confirm the discriminatory value of these two ASVs and the impact of sex on microbial richness we used regression analyses. After adjusting for HIV status, age, BMI/A and recent antibiotic use, associations between sex and microbial richness, Firmicutes and *Bacteroides* were lost (**Supplementary Table 9**).

### 3.6 Proteobacteria are associated with CD4+ T cell activation and exhaustion

**Figure 2:**
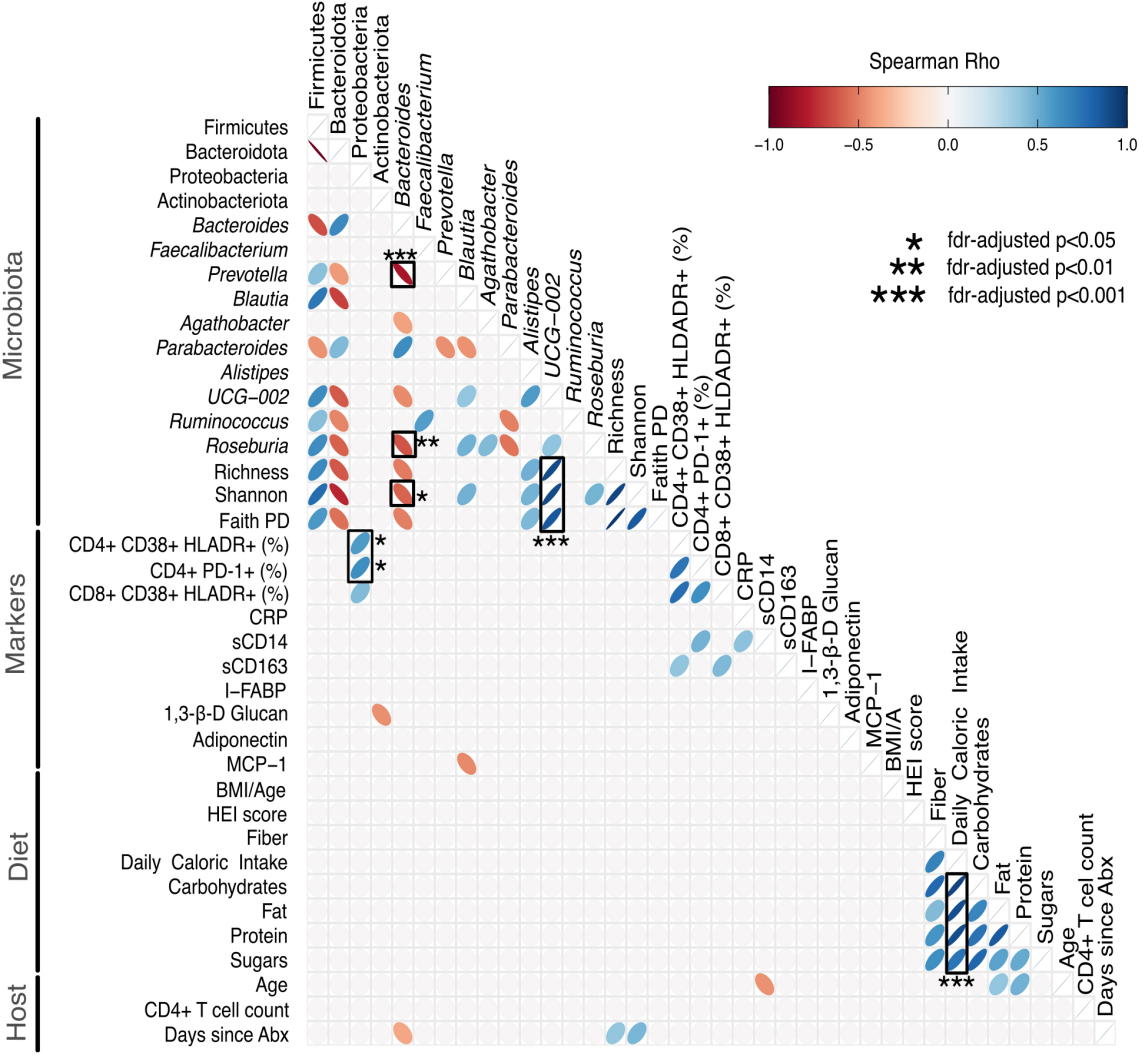
Heatmap showing relationships between the gut microbiota, markers of HIV disease progression/pathogenesis, diet and host metrics. Correlations were performed using nonparametric Spearman, and p values were adjusted for multiple correlations using the false discovery rate (FDR). We included the 4 predominant phyla (Firmicutes, Bacteroidota, Proteobacteria, and Actinobacteria), the top 10 genera, alpha diversity metrics, markers linked to HIV disease progression and pathogenesis, diet and host metadata. Correlations with an unadjusted p>0.05 were removed for ease of visualization (empty cells). Significant FDR-adjusted p values are marked with a black box, and the level of significance (* FDR-adjusted p<0.05, ** FDR-adjusted p<0.01, *** FDR-adjusted p<0.001) is provided. Positive correlations are shown in blue, and negative correlations are shown in red. The heatmap was created using ggcorrplot2 version 0.1.2 in R. %, percentage; Abx, antibiotics; β, beta; BMI/A, body mass index for age; CD, cluster of differentiation; CRP, C-reactive protein; FDR, false discovery rate; I-FABP, intestinal fatty acid binding protein; HEI, healthy eating index; MCP-1, monocyte chemoattractant protein-1; PD, phylogenetic distance; PD-1, programmed cell death-1; s, soluble.

Finally, we related the gut microbiota to markers of gut health, HIV disease progression and pathogenesis, dietary patterns and host metadata. Relationships were explored by nonparametric correlation to provide an overview of microbiota-diet-host associations. We limited the number of correlations and included the 4 predominant phyla (Firmicutes, Bacteroidota, Proteobacteria, and Actinobacteria), the top 10 genera, alpha diversity, most markers linked to HIV disease progression and pathogenesis, diet-related markers (BMI/A, HEI score, daily caloric intake, 3 macronutrients, sugar) and host metadata (age, immune status, antibiotic usage). Correlations were performed using nonparametric Spearman, and p values were corrected [false discovery rate (FDR)] as shown in **Figure 2**. We found strong correlations (positive and negative) among Firmicutes, Bacteroidota, and their respective genera, and between Firmicutes, Bacteroidota (and their respective genera) and alpha diversity. In particular, the genus *UCG-002* was positively correlated with all 3 indices of alpha diversity (rho>0.8, FDR-adjusted p<0.001 for all 3 correlations). *Bacteroides* was inversely correlated with *Prevotella (*rho=-0.822, FDR-adjusted p<0.001) and *Roseburia* (rho=-0.63, FDR-adjusted p=0.0084) and Shannon (rho=-0.587, FDR-adjusted p=0.025). Both the frequency of activated CD4+ T cells (as measured by CD38+ HLADR+) and exhausted CD4+ T cells (as measured by PD-1+) were positively correlated with Proteobacteria (rho=0.568. FDR-adjusted p= 0.029, and rho=0.62, FDR-adjusted p=0.0126, respectively).

## 4 Discussion

The work presented here characterized the gut microbiota in CLWH, and assessed its relation to markers of HIV disease progression and pathogenesis (and others), dietary patterns and host metadata, comparing it with HUU. We found no clear evidence that HIV impacted the gut microbiota composition, structure or diversity, nor did we find discriminant taxa between CLWH and HUU. Our results are both in agreement and in contrast with previously published studies in CLWH. Kaur and colleagues (22) showed that alpha diversity was decreased in CLWH compared to uninfected children, in both CLWH naïve to ART and CLWH on ART. CLWH naïve to ART had the least alpha diversity. They also reported that CLWH had a distinct pattern of gut microbiota compared to uninfected children, characterized by increased Bacteroidetes, and decreased Firmicutes, and Actinobacteria at phylum level, and increased *Prevotella*, and decreased *Bacteroides*, and *Ruminococcus* at genus level. Abange and colleagues (24) also reported that CLWH had lower alpha diversity and altered beta diversity. They found that CLWH had lower relative fractions of butyrate producers and obligate anaerobes, that *Prevotella* was decreased, together with gammaproteobacteria (*Haemophilus influenzae*) and Enterobacteriaceae, while *Akkermansia* and *Faecalibacterium* were increased. In contrast, Flygel and colleagues (25) suggested that prolonged ART (> 10 years) might restore microbial richness in CLWH. Flygel and colleagues also reported increased beta-diversity and a rectal microbiota enriched in 3 genera from the phylum Actinobacteria (*Corynebacterium*, *Lawsonella*, *Collinsella*) and 4 genera from the phylum Firmicutes (*Finegoldia*, *Anaerococcus*, *Erysipelotrichaceae*, and *Lachnoclostridium*). Sessa and colleagues (46) reported higher alpha diversity in CLWH, and two distinct clusters characterized by *Akkermansia muciniphila* in cluster 1 and *Bacteroides*, *Faecalibacterium* (and others) in cluster 2. Goosen et al. (26), reported that alpha diversity did not differ between CLWH and uninfected controls, while beta diversity was significantly higher in CLWH. Finally, Tincati and colleagues (27) found similar bacterial microbiota composition and alpha diversity in fecal samples. They also interrogated the plasma microbiome and found no difference in alpha and beta diversity. However, they did find a predominance of Gammaproteobacteria and *Pseudomonas* spp. in HIV-exposed infected compared to HIV-exposed uninfected children.

Discrepancies between studies are expected. These could be due to differences in the number of participants included (small versus large cohorts), age of children, inclusion of CLWH naïve to ART, or only CLWH on ART, specific ART regimens and length of ART duration, whether CLWH on ART were virally suppressed or viremic, use of antibiotics, and the choice of HIV-uninfected controls [whether HIV-unexposed and uninfected or HIV-exposed and uninfected (27)]. Furthermore, other variables including geographic location and even ethnic groups within the same country (24) are also likely to impact results, precluding their generalization. Kaur and colleagues included both CLHW naïve to ART and on suppressive ART and compared them to age- and sex-matched uninfected children. In this cohort, children were excluded if antibiotics were used one month prior to sample collection. Abange and colleagues included CLWH on ART, predominantly NNRTI-based regimen, for a median of 5 years and non-exposed HIV-negative children. Some CLWH were viremic (n=13) and most had CD4>500 cells/mm^3^. Flygel and colleagues investigated the gut microbiota in CLWH on long-term ART (most CLWH had chronic lung disease) and HIV-uninfected children, and used rectal swabs instead of fecal samples. Sessa and colleagues included aviremic CLWH for a median of 9 years (46). Goosen and colleagues (26) included aviremic CLWH aged 8 to 13 years, and excluded children with recent antibiotic use, and those with BMI/A <-3 or >2. Finally, Tincati and colleagues (27) included HIV-exposed infected children all on ART but not all aviremic, and HIV-exposed uninfected children (born from HIV-infected mothers), and excluded children with previous antibiotic exposure (2 weeks), gastrointestinal disorders and co-infections. Compared to other studies, our cohort included a small number of participants, CLWH were all aviremic, on PI-based ART for the most part. Interestingly, Abange and colleagues (24) reported that CLWH on PI-based regimen had lower alpha diversity and altered composition when compared to CLWH on NNRTI, as well as their HIV non-infected counterparts. In particular, they found that *Blautia*, *Prevotella*, *Oscillospira*, and *Faecalibacterium* were more abundant in NNRTI compared to PI/r, with CLWH on PI/r had a lower abundance of microorganisms predicted to be butyrate producers and obligate anaerobes. Given the predominant use of PI-based regimen in our cohort and the small number of CLWH, subgroup analysis looking at the potential impact of ART on the gut microbiota was not possible. Interestingly, we reported differences in the gut microbiota of Mexican ALWH on PI/r-based and EFV-based regimen (36). Although alpha diversity was not significantly different between ART regimens, we did find higher microbial translocation (assessed by plasma sCD14) and enterocyte damage (assessed by plasma levels of I-FABP) in those ALWH on PI/r-based regimen. Flygel and colleagues found no association between beta diversity and type of ART, nor years on ART (25). Both studies were however in agreement with the fact that duration of ART was not associated with alpha and beta diversity, suggesting that in pediatric HIV and viremia were not predictive of microbiota diversity (12), in contrast with ALWH (47, 48).

Some studies, including ours, suggest a beneficial impact of long-term ART on the gut microbiota. Some genera associated with intestinal health were also found in CLWH [*Akkermansia*, *Faecalibacterium*, (24)]. In agreement with other studies (25–27), we found that long-term ART might restore all metrics of alpha diversity. The median time on ART was similar between these studies and ours. We found no differences in alpha and beta diversity, and a high abundance of *Faecalibacterium*. *Akkermansia* was not among the top 20 genera in our cohort, but we were able to detect this genus, the mean overall abundance was 0.2% in CLWH and 0.7% in HUU. *Faecalibacterium* (Firmicutes) and *Akkermansia* (Verrucomicrobia) are two genera thought to be beneficial, due to their protective role in metabolism, immune system and gut barrier (49). Kaur and colleagues found decreased abundance of *Faecalibacterium* in CLWH on ART compared to HIV-uninfected controls (22). Interestingly, Sessa and colleagues found that the gut microbial group represented predominantly by *Akkermansia* (*A. muciniphila*) was linked to higher markers of inflammation, vascular endothelial activation, microbial translocation, but not T cell activation (46). Their results suggest that overabundance of a beneficial commensal could have negative implications. Indeed, *Akkermansia* has been linked to deleterious effects (50).

We report that markers linked to HIV disease progression and pathogenesis were similar to those of HUU, which suggests that prolonged ART might normalize their levels. Our results are in agreement with Fitzgerald and colleagues (51) who found that immune activation decreased in pediatric HIV-infected children after ART initiation. Weinberg and colleagues also found that several markers including IFN-, IL-6, TNF-, sCD163, CRP, sCD14 and MCP-1 decreased after virologic control (52). Others found that ALWH on stable ART showed evidence of altered gut permeability and fungal translocation (53) and increased monocyte and T cell activation and higher sCD14 (54). In our study, no differences in inflammation, immune activation, bacterial and fungal translocation, and enterocyte damage were observed between CLWH and HUU. However, our results also differ from Tincati and colleagues (27). They found elevated levels of sCD14, IL-6 in HIV-exposed infected children compared with HIV-exposed uninfected children, however levels of I-FABP were similar. They raise an important point in their discussion regarding the choice of the control uninfected group used to compare with HIV-exposed infected children, as we alluded to earlier. Most studies enroll HIV-unexposed and uninfected children. As shown in ALWH, careful consideration is needed to enroll the most appropriate control group (55). Studies in perinatal HIV should ideally include both HIV-exposed uninfected and HIV-unexposed uninfected children, when possible. We did find increased frequency of exhausted T cell, a marker of HIV disease progression in CLWH. This is in agreement with other studies in CLWH (56, 57). Exhausted T cells are also a marker of disease progression in ALWH (58). In CLWH, PD-1+ T cells are heterogenous population of central and effector memory T cells that have impaired proliferative capacity and secrete proinflammatory cytokines. Interestingly, we also found that Proteobacteria, a phylum known to harbor pathobionts, was associated with the frequency of activated CD4+ T cells and exhausted CD4+ T cells, linking the gut microbiome to persistent immune exhaustion and premature aging. In our cohort, the mean overall abundance of Proteobacteria was 2.40%, when stratifying by HIV, we found an increase in CLWH (2.8%) compared with HUU (2.1%). At genus level, we found an unclassified Alphaproteobacteria, *Sutterella* (order of Burkholderiales) and *Escherichia-Shigella* (family Enterobacteriaceae), known to translocate and elicit pro-inflammatory responses (59, 60). Interestingly, Tincati and colleagues (27) found that the plasma microbiome was enriched in Gammaproteobacteria and *Pseudomonas* spp. in HIV-exposed infected children and Alphaproteobacteria in HIV-exposed uninfected children. They also reported a weak trend towards an inverse correlation between plasma *Pseudomonas* spp. and CD4+ T cell counts. Collectively, our results suggest that although some markers and gut microbiota metrics might normalize or differences may not be apparent or quantifiable (in particular if studies are underpowered like ours), CLWH are at increased risk of premature aging. Also, the CD4/CD8 ratio did not normalize, which is concordant with the fact that most CLWH in our study started ART later. Our findings could also be attributable, at least in part to the inclusion of CLWH and HUU who had recently taken antibiotics. Interestingly, studies have shown that co-trimoxazole increased alpha diversity (23), decreased inflammation (61) and suppressed the gut-resident viridans group streptococcal species (VGS) associated with intestinal inflammation (62). At the same time, antibiotics are a confounder in gut microbiota studies (63) and have long-term adverse effects of the gut microbiota (32). Most studies report that antibiotic use causes a reduction in microbial diversity and richness, including a decrease in beneficial bacterial taxa, resulting in metabolic alterations, increased susceptibility to colonization by pathogens and an increase in bacterial resistance to antibiotics. Children in this study (CLWH and HUU alike) reported a high number of antibiotics courses, even considering the recall bias that parents might have had at the time of the interview. Antibiotics taken by children in this study were primarily β-lactams, broad-spectrum antibiotics which interrupt bacterial cell-wall formation (64). When assessing the impact of antibiotics on the gut microbiota, we found none. Anecdotally, stool consistency was not indicative of diarrhea associated with antibiotics (type 6-7), most children had normal stool consistency (type 3-4). These results were unexpected as the cumulative effects on the microbiome as a result of repeated administration of multiple courses of antibiotics have been demonstrated^29,30^. Consequences have also been reported with children facing the risk of experiencing temporary or chronic alterations in their microbiome, which could predispose them to develop chronic disorders ^27,28^. Our findings could be attributed, at least in part, to the fact that amoxicillin-clavulanate was the most frequently prescribed antibiotic, and it has been reported to cause no or only mild to moderate changes in the composition of the intestinal microbiome (65, 66).

Diet is one of the key modulators of the gut microbiota (63). In this study, children had a diet that is representative of the Mexican urban population (67). Both diet and its effect on the gut microbiota have clearly been linked to obesity, metabolic syndrome, and type 2 diabetes (68, 69). We found no associations between dietary intake amounts or diet quality (HEI score) and gut microbiota metrics in the context of HIV infection. We also found that markers of adipose tissue inflammation were comparable between CLWH and HUU. Generoso and colleagues also found that sCD163 normalized in Kenyan CLWH on ART (70). According to the Encuestas Nacionales de Salud y Nutrición (Ensanut, National Health and Nutrition Surveys), Instituto Nacional de Salud Pública (INSP, National Institute of Health) in 2018-2019, the prevalence of overweight and obesity in children aged 5 to 11 years was 18.1 and 17.5% (respectively) and in adolescents aged 12 to 19 years was 23.8 and 14.6%, respectively (71). In our study, no CLWH had obesity, compared to 2 HUU who had obesity (no significant differences were observed between groups). Childhood obesity is a growing health problem worldwide including Mexico (72). Childhood obesity in the context HIV infection is further alarming given the increased prevalence of cardiometabolic diseases and other NCD. Improving the health of CLWH (and HUU) is very important. One possible avenue is dietary interventions, which can be very successful short-term, but are not largely sustainable over time (73, 74). Food and nutrition literacy should only be explored, as empowering children with knowledge and skills related to nutrition and dietary habits, are likely to promote healthier eating behaviors from an early age (75). Interventions targeting the gut microbiota using different approaches have had relatively little success so far (13, 76). A recent study reported an attenuation of bacterial dysbiosis in vertically HIV-infected children (n=24), randomized to receive a daily nutritional supplementation or placebo for 4 weeks (77). Authors postulated that the gut microbiota is less resilient in children and better amiable to change and therefore would respond better to interventions. Interventions to promote a healthier gut microbiome, together with healthier lifestyle to increase life expectancy, quality in CLWH is necessary as a pediatric HIV cure is still lacking (78). CLWH will face many challenges as they transition to adulthood, and efforts should be made to minimize NCD and other comorbidities, which will negatively impact their quality of life. Interestingly, no correlations were found between bacterial genera, alpha diversity and dietary variables. We did find that alpha diversity metrics were positively correlated with *UCG-002*, a genus of the Oscillospiraceae family. *UCG-002* has been negatively associated with obesity and insulin resistance, suggesting that *UCG-002* may be involved in mechanisms that improve insulin sensitivity (79, 80). Another study reported an enrichment of *UCG-002* in pubertal participants, as well as an increased abundance of *Bacteroides* and *Prevotella* in individuals at different stages of puberty (81). The authors suggest that these bacterial genera may represent a more mature stage of the gut microbiota. It is therefore plausible, though highly speculative, that the positive association between *UCG-002* and alpha diversity may indicate, at least in part, the start of the transitional change from childhood to adulthood, impacting the gut microbiota. The mean of our cohort was 9.53 years.

We found that sex impacted the gut microbiota with female children being *Bacteroides* predominant and having lesser microbial richness compared to their male counterparts, although after adjusting for HIV status, age, BMI/A and recent antibiotic use, these associations were lost. Recent studies have shown that sex (and sex hormones) contribute to differences in the gut microbiome (82), as early as birth and throughout life. Interestingly, differences in inflammation profiles by sex have also been reported (52, 83). Because our primary objective was to understand the impact of HIV infection, associations between markers of HIV disease progression and sex or age were not explored. Differential effects of age have also been reported (84). We acknowledge that this is a limitation. In HIV infection, *Bacteroides* is a common enterotype, together with *Prevotella*, this latter is linked to sexual preference in ALWH (55). Bacteroides abundance has also been linked to consumption of protein- and animal-fat rich diets (85) and an increased *Prevotella*/*Bacteroides* has been linked to obesity (86). Interestingly, a study in Mexican children also reported the *Bacteroides* was highly abundant in children with type 1 diabetes (87). Additionally, we found a negative correlation between *Prevotella* and *Bacteroides*, which has been previously reported (86, 88). Our understanding of which variables are responsible for these taxonomic shifts remains incomplete. Indeed, Sessa and colleagues (46) failed to identify clinical variables that would explain the presence of two bacterial clusters in CLWH (*A. muciniphila*, less diverse, and *Bacteroides* and other genera, more diverse). Factors that impact the gut microbiota are likely to have synergistic and cumulative effects, making teasing out the impact of each factor difficult. Disentangling these effects are very important in the immediate future to improve our understanding of HIV-associated microbial dysbiosis.

This study has several limitations. First, our cohort included a small number of participants, and is underpowered to detect differences. Also, our cohort was not matched on age. The inability to balance groups on confounders might have introduced bias in our analyses. Second, CWH and HUU were recently exposed to antibiotics, which might have masked differences in bacterial community structure. Also, although our results suggest microbial signatures linked to CD4+ T cell activation and exhaustion (Proteobacteria) and sex (*Bacteroides*), these should be interpreted carefully given the high degree of interindividual variation in gut microbiota, which are not accurately represented when visualizing data (mean relative abundance) using heatmaps or barplots. Also, we were limited to reporting taxonomic classification to genus level, a limitation inherent to the16S targeted sequencing. Subgroup analyses to explore the impact of different variables (ART, diet) were not possible due to low number of participants in each subgroup. Finally, our study is cross-sectional and does not allow to establish causality. At the same time, our study also has strengths, as we included a comprehensive use of biologics: dietary patterns and quality; several markers of inflammation, immune activation (soluble and cellular), microbial and fungal translocation, enterocyte integrity, and adipose tissue inflammation, host metadata and explored relationships with the gut microbiota.

## 5 Conclusion

Given the prolonged exposure to HIV and ART, and prophylactic exposure to antimicrobials, the gut microbiota of CLWH may be less resilient, increasing tolerance to pathobionts. This, in turn, may lead to increased prevalence of cardiometabolic alterations, and NCD, or their premature symptomatic presentation compared to the general population. Here we report that the gut microbiota of CLWH appears similar to that of HUU, and that most markers of HIV disease progression normalize on ART. Our findings suggest that long-term ART administration (8 years) in CLWH, might have a beneficial impact on the gut microbial ecology and immune activation/inflammation. Sex is also likely to impact the gut microbiota. CLWH do exhibit signs of premature aging (frequency of exhausted CD4+ T cells), which puts them at risk for increased comorbidities in adulthood. Interventions to reverse HIV-associated premature aging, in synergy with interventions targeting overweight and obesity are likely to positively impact the quality of life and longevity of CLWH.

## Data availability statement

The datasets presented in this study can be found in online repositories. The names of the repository/repositories and accession number(s) can be found below: PRJNA868762 (SRA).

## Ethics statement

The studies involving humans were approved by Ethics and Scientific committees of INER and IMSS. The studies were conducted in accordance with the local legislation and institutional requirements. The human samples used in this study were acquired from the recruitment of participants to a research protocol. Written informed consent and age-appropriate assent were obtained from all participants and/or parents before study enrollment.

## Author contributions

VB-M, SP-C, CR-P: Designed the study. CR-P, NAMJ, HP-L, JV-R: Recruited participants. CR-P: performed interviews and questionnaires. CR-P, IO-P: Performed dietary assessments. CA-A: Performed laboratory determination of lymphocyte populations. MC-T, NM, SP-C: Performed laboratory experiments. SP-C, CR-P: Analyzed the data. VB-M, GR-T, SA-R: Provided critical intellectual contributions. GRT, VB-M, SP-C: Funding acquisition. CR-P, SP-C: Wrote the manuscript with contributions from all co-authors. All authors approved the final version of this manuscript.
