# Supplementary material for: Distinct fecal microbial signatures are linked to sex and chronic immune activation in pediatric HIV infection
Source: Front Immunol. 2023 Aug 29;14:1244473. doi: 10.3389/fimmu.2023.1244473 (PMC10497879; doi:10.3389/fimmu.2023.1244473)
Supplement: Supplementary file 1 [file DataSheet_1.pdf]

Supplementary Material

Distinct fecal microbial signatures are linked to sex and chronic immune activation in pediatric HIV infection

Cecilia Rosel-Pech[1,2 †], Sandra Pinto-Cardoso[3†*], Monserrat Chávez-Torres[3], Nadia Montufar[3], Iván Osuna-Padilla[3], Santiago Ávila-Ríos[3], Gustavo Reyes-Terán[1], Charmina Aguirre-Alvarado[2], Norma Matías-Juan[4], Héctor Pérez-Lorenzana[5], José Guillermo Vázquez-Rosales[6], Vilma Carolina Bekker-Méndez[2*]

*** Correspondence:**
Corresponding authors
Sandra Pinto-Cardoso, PhD
Centro de Investigación en Enfermedades Infecciosas,
Instituto Nacional de Enfermedades Respiratorias Ismael Cosío Villegas,
Ciudad de México, México
Electronic address: sandra.pintocardoso.cieni@gmail.com

Vilma Carolina Bekker-Méndez, PhD
Unidad de Investigación Médica en Inmunología e Infectología,
Centro Médico Nacional La Raza,
Instituto Mexicano del Seguro Social,
Ciudad de México, México
Electronic address: bekkermendez@yahoo.com

Supplementary Table 1. Monoclonal antibodies used in this study

Marker	Population of interest	Fluorochrome	Clone	Supplier
CD3	Lymphocytes	BV570	UCHT1	Biolegend
CD4	Lymphocytes	APC-Cy7	A161A1	Biolegend
CD8	Lymphocytes	AF700	RPA-T8	BD Pharmingen
CD38	Activation	BV711	HIT2	Biolegend
HLADR	Activation	BV785	L243	Biolegend
CD57	Terminal differentiation	APC	HCD57	Biolegend
PD-1	Regulator of T-cell exhaustion	BV605	EH12.2H7	Biolegend
Aqua Dye	Live cells	BV510	NA	Invitrogen
Ki67	Cycling	BV421	Ki67	Biolegend
CD19	B cells	BV510	HIB19	Biolegend
CD14	Monocytes	BV510	M5E2	Biolegend
CD56	NK cells	BV510	HCD56	Biolegend
CD11c	Dendritic cells	BV510	3.9	Biolegend
CD123	Dendritic cells	BV510	6H6	Biolegend

Abbreviations: CD: cluster of differentiation, CCR: C-C chemokine receptor, PD-1: Programmed cell death protein-1, HLA: Human Leukocyte Antigen, NK: natural killer

Supplementary Figure 1. Representative gating strategy

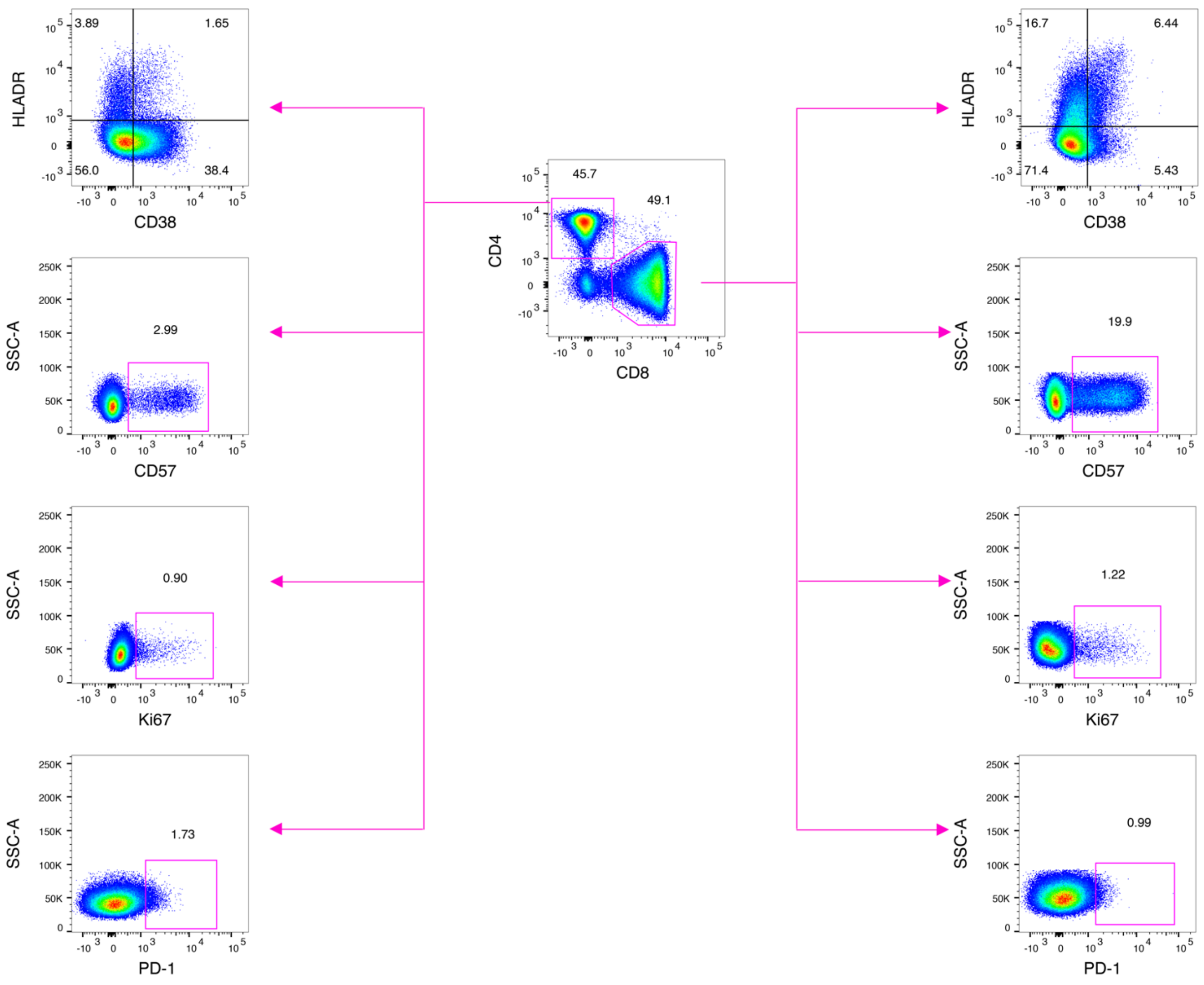

Supplementary Figure 1. A representative gating strategy is shown. Peripheral blood mononuclear cells were thawed and stained for extracellular (CD3, CD4, CD8, CD38, HLADR, CD57, PD-1) and intracellular (Ki67) markers are described in Methods. PBMCs from all samples were thawed and stained the same day. Fluorescence minus one (FMO) for all fluorophores in this panel were used to accurately set the gates for positive populations. Flow cytometry acquisition was performed on a BD LSRFortessa (BD Biosciences, San Jose, CA, USA) within 24 h of staining. Quality controls were performed using BD cytometer Setup & Tracking Beads and rainbow beads (BD biosciences). A compensation matrix was calculated and applied using BD Comp Beads (BD Biosciences). A minimum of 3 million events were acquired (mean of 3,701,250 events). Data was analyzed using FlowJo™ v10.9 (BD, Ashland, Oregon, Unites States). Raw FCS files were first quality-controlled using FlowAI v2.3.1 using default parameters, except for the changepoint penalty (set to 500). A mean of 2,529,571 events were retained after quality-control. Populations were pre-gated for singlets and morphology by using forward versus side scatter, followed by the exclusion of dead cells (aqua dye negative events) and unwanted populations (CD14, CD19, CD11c, CD123, CD56, dump gate). Live cells were gated on CD3+ and on CD4+ or CD8+ T-cells.

Abbreviations:
CD: cluster of differentiation, PD-1: programmed cell death protein-1, SSC-A: side scattered area

Supplementary Table 2. HIV clinical parameters of CLWH at HIV diagnosis and at study enrollment (n=15)

HIV clinical parameters	CLWH
**At HIV diagnosis**	
Age (years)	2 (0.4-3)
Plasma viral load (copies/mL)	84550 (17380-419000)
CD4 T cell count (cells/µL)	623 (515-812)
CD8 T cell count (cells/µL)	1232 (734-2114)
CD4/CD8 ratio	0.49 (0.27-0.89)
**Time between**	
Birth and HIV diagnosis (years)	1.67 (0.3-3.5)
HIV diagnosis and ART initiation (days)	12 (3-53)
HIV diagnosis and study enrollment (years)	8 (6-9)
ART initiation and study enrollment (years)	8.3 (7.5-10.6)
**At study enrollment**	
Plasma viral load	
Undetectable* (<200 copies/mL)	15 (100)
CD4 T cell count (cells/µL)	905 (718-1237)
ART regimen	
PI	10 (66.67)
InSTI	4 (26.67)
NNRTI	1 (6.67)

Data are expressed as median (interquartile range) or number (percentage) as appropriate.
*All CLWH had undetectable plasma viral load (<40 copies/mL), except one CLWH had a pVL of 122 copies/mL.

Abbreviations
ART: antiretroviral therapy, CLWH: children living with HIV, HIV: human immunodeficiency virus, IQR: interquartile range, InSTI: integrase strand transfer inhibitors, mL: milliliter, µL: microliter, NNRTI: non-nucleoside reverse transcriptase inhibitors, NRTI: nucleoside reverse transcriptase inhibitors, PI: protease inhibitors

Supplementary Table 3. Dietary assessment based on food frequency questionnaire: overall and stratified by HIV status

Macronutrients	All	CLWH	HUU	Unadjusted P value*
Daily caloric intake (kcal)	1527.83 (1316.80-1936.5)	1714.27 (1394.23-2029.53)	1459.98 (1262.35-1838.32)	0.202
Total protein (g)	76.07 (51.93-93.47)	77.50 (60.63-94.10)	57.53 (48.87-85.17)	0.054
Vegetable protein (g)	2.0 (1.23-3.50)	2.30 (0.50-3.50)	1.83 (1.57-3.48)	0.711
Animal protein (g)	72.57 (50.70-89.13)	74.80 (61.73-94.10)	55.54 (46.77-83.88)	**0.037**
Total fat (g)	53.00 (37.63-65.57)	53.00 (37.63-80.40)	54.45 (40.33-62.93)	0.892
Polyunsaturated fatty acids (g)	11.50 (6.70-16.30)	12.30 (5.93-16.37)	11.18 (7.05-12.75)	0.770
Monounsaturated fatty acids (g)	15.96 (13.75-20.23)	17.9 (14.56-21.20)	15.25 (13.19-19.21)	0.202
Saturated fat (g)	17.60 (14.07-23.93)	16.37 (14.60-26.37)	18.38 (13.37-23.65)	0.770
Cholesterol (mg)	207.83 (168.03-279.27)	207.30 (178.87-284.70)	228.98 (141.85-270.70)	0.770
Carbohydrates (g)	210.60 (159.53-250.70)	221.10 (177.13-277.60)	194.30 (148.50-239.18)	0.247
Sugars (g)	16.95 (11.77-38.60)	30.83 (11.77-61.40)	15.48 (11.95-29.87)	0.216
Fiber (g)	16.93 (13.65-22.93)	17.13 (15.57-25.30)	16.40 (12.91-18.90)	0.318
**Micronutrients**				
Vitamin B1 (mg)	1.20 (0.93-1.77)	1.60 (1.10-1.90)	1.00 (0.90-1.37)	**0.049**
Vitamin B2 (mg)	1.67 (1.20-2.03)	1.80 (1.20-2.03)	1.60 (1.03-2.05)	0.358
Vitamin B6 (mg)	1.73 (1.23-2.57)	2.10 (1.57-3.00)	1.47 (1.15-2.03)	**0.030**
Folic acid (µg)	263.07 (226.00-375.00)	276.53 (226.00-449.93)	251.67 (224.33-371.17)	0.545
Vitamin A (µg)	636.90 (407.93-937.00)	599.07 (391.13-937.00)	647.05 (504.63-904.95)	0.682
Carotene (mg)	0.77 (0.30-2.10)	0.77 (0.30-2.10)	0.82 (0.28-2.05)	0.922
Vitamin E (mg)	2.40 (1.67-3.17)	2.03 (1.27-3.17)	2.53 (1.82-3.23)	0.423
Vitamin C (mg)	73.47 (50.93-116.73)	69.17 (46.27-128.67)	85.73 (56.87-109.25)	0.892

Sodium (mg)	1372.23 (1051.15-1904.03)	1554.57 (1192.10-2324.93)	1320.80 (925.37-1732.45)	0.338
Potassium (mg)	2146.83 (1896.63-2426.63)	2313.30 (1910.50-3255.27)	1946.58 (1721.15-2291.45)	0.086
Calcium (mg)	791.80 (604.80-872.10)	827.10 (668.30-909.73)	770.45 (578.02-841.62)	0.202
Magnesium (mg)	249.90 (207.13-318.20)	287.80 (223.07-381.90)	238.40 (197.97-274.53)	0.054
Phosphorus (mg)	1278.53 (979.00-1457.37)	1395.17 (1190.40-1782.67)	1086.42 (932.37-1313.42)	**0.027**
Iron (mg)	10.90 (8.73-16.03)	12.93 (9.47-17.17)	9.66 (8.63-13.87)	0.119
Zinc (mg)	8.50 (6.27-10.67)	9.87 (7.10-11.30)	7.22 (6.11-10.08)	0.086

Data are presented using median and interquartile range (IQR). Wilcoxon Rank Sum Test was used to compare CLWH and HUU.

*Only unadjusted p values are shown. When adjusting p values for multiple comparisons using false discovery rate (FDR), all significant p values are lost. Statistical significance was considered with $p<0.05$.

Supplementary Table 4. Dietary assessment based on three 24-hour recalls: overall and stratified by HIV status

**Macronutrients**	**All**	**CLWH**	**HUU**	**Unadjusted P value***
Daily caloric intake (kcal)	2175.47 (1574.53-2503.33)	2378.97 (1943.33-2696.00)	1858.52 (1334.90-2301.30)	0.060
Total protein (g)	80.97 (59.38-102.66)	92.57 (66.70-109.61)	70.27 (56.08-91.68)	0.140
Vegetable protein (g)	6.81 (5.47-8.84)	7.12 (5.97-9.50)	6.25 (5.28-8.50)	0.216
Animal protein (g)	73.06 (55.40-93.82)	87.11 (58.78-103.60)	63.26 (50.00-86.92)	0.119
Total fat (g)	67.18 (55.58-83.73)	77.13 (62.64-88.66)	59.92 (39.23-81.14)	0.078
Polyunsaturated fatty acids (g)	12.71 (10.20-15.77)	14.50 (12.71-16.63)	10.33 (7.79-13.13)	**0.017**
Monounsaturated fatty acids (g)	22.72 (16.45-26.15)	24.85 (20.3-28.16)	19.41 (13.95-24.04)	0.060
Saturated fat (g)	20.32 (13.99-28.44)	22.84 (18.92-29.19)	18.18 (11.68-26.95)	0.140
Cholesterol (mg)	247.22 (185.13-330.39)	295.10 (208.77-367.57)	191.35 (163.70-278.40)	**0.033**
Carbohydrates (g)	303.28(189.79-352.13)	333.53 (288.63-369.90)	247.70 (167.61-330.06)	0.086
Sugars (g)	53.85 (40.29-84.29)	79.52 (47.31-101.06)	50.04 (38.46-69.40)	0.119
Fiber (g)	29.65 (19.08-36.74)	30.99 (21.09-37.24)	28.13 (17.97-34.55)	0.599
**Micronutrients**				
Vitamin B1 (mg)	1.74 (1.13-2.50)	1.74 (1.28-2.51)	1.76 (1.11-2.33)	0.953
Vitamin B2 (mg)	2.13 (1.32-2.87)	2.06 (1.45-2.94)	2.26 (1.21-2.72)	0.830
Vitamin B6 (mg)	2.61 (2.20-3.78)	2.61 (2.29-4.43)	2.82 (1.98-3.67)	0.830
Folic acid (µg)	413.83 (277.91-544.70)	438.13 (337.73-544.70)	404.97 (268.65-516.84)	0.470
Vitamin A (µg)	1554.07 (957.00-2021.90)	1554.07 (957.00-1965.90)	1598.75 (845.95-2099.99)	0.892
Carotene (mg)	4.24 (2.22-7.06)	4.10 (1.62-7.11)	4.54 (2.83-6.98)	0.800
Vitamin E (mg)	6.97 (5.03-8.43)	6.82 (6.09-8.43)	7.04 (4.20-8.06)	0.626
Vitamin C (mg)	199.56	192.62	227.75	0.520

	(152.94-303.38)	(152.09-246.00)	(152.98-319.89)	
Sodium (mg)	1348.87 (1018.30-1719.13)	1454.63 (1336.00-2042.63)	1048.57 (744.32-1475.83)	**0.007**
Potassium (mg)	3600.00 (2588.30-4506.00)	3600.00 (3155.70-4533.33)	3627.00 (2214.98-4252.34)	0.446
Calcium (mg)	1020.17 (759.43-1455.33)	1020.17 (849.00-1521.10)	1026.13 (657.21-1326.04)	0.423
Magnesium (mg)	398.10 (251.19-478.13)	403.43 (332.36-488.70)	381.12 (218.35-448.64)	0.188
Phosphorus (mg)	1419.43 (1047.10-1896.10)	1462.43 (1361.57-2017.67)	1356.65 (911.82-1746.70)	0.101
Iron (mg)	17.46 (12.28-43.43)	27.23 (13.70-107.78)	16.56 (11.24-30.39)	0.338
Zinc (mg)	10.57 (8.24-13.76)	10.72 (9.32-15.05)	9.23 (6.49-12.29)	0.151

Data are presented using median and interquartile range (IQR). Wilcoxon Rank Sum Test was used to compare CLWH and HUU.
*When adjusting p values for multiple comparisons using false discovery rate (FDR), all significant p values are lost.
Statistical significance was considered with $p<0.05$.

Supplementary table 5. Association between HIV status and the frequency of exhausted CD4+ T cells (PD-1+)

	**Univariate analysis**		**Adjusted for sex, age, and BMI**		**Adjusted for sex, age, BMI, and recent antibiotic use**	
	β coefficient (95% CI)	p value	β coefficient (95% CI)	p value	β coefficient (95% CI)	p value
CD4+ PD-1+ (%)	0.70 (0.19-1.21)	**0.0083**	0.93 (1.46-4.41)	**0.0019**	0.99 (0.391.59)	**0.0024**

Linear regression analysis between the outcome variable (% of CD4+ PD-1+) and HIV status (reference: CLWH. The univariate model was adjusted for sex, age and BMI/A (model 1), and sex, age, BMI/A and recent antibiotic use (antibiotics used in the 3 previous months) (model 2).

Abbreviations: CI: %: percentage, BMI: body mass index, CI: confidence interval, PD-1: programmed cell death-1

Supplementary Figure 2. No discernable impact of HIV on the gut microbiota

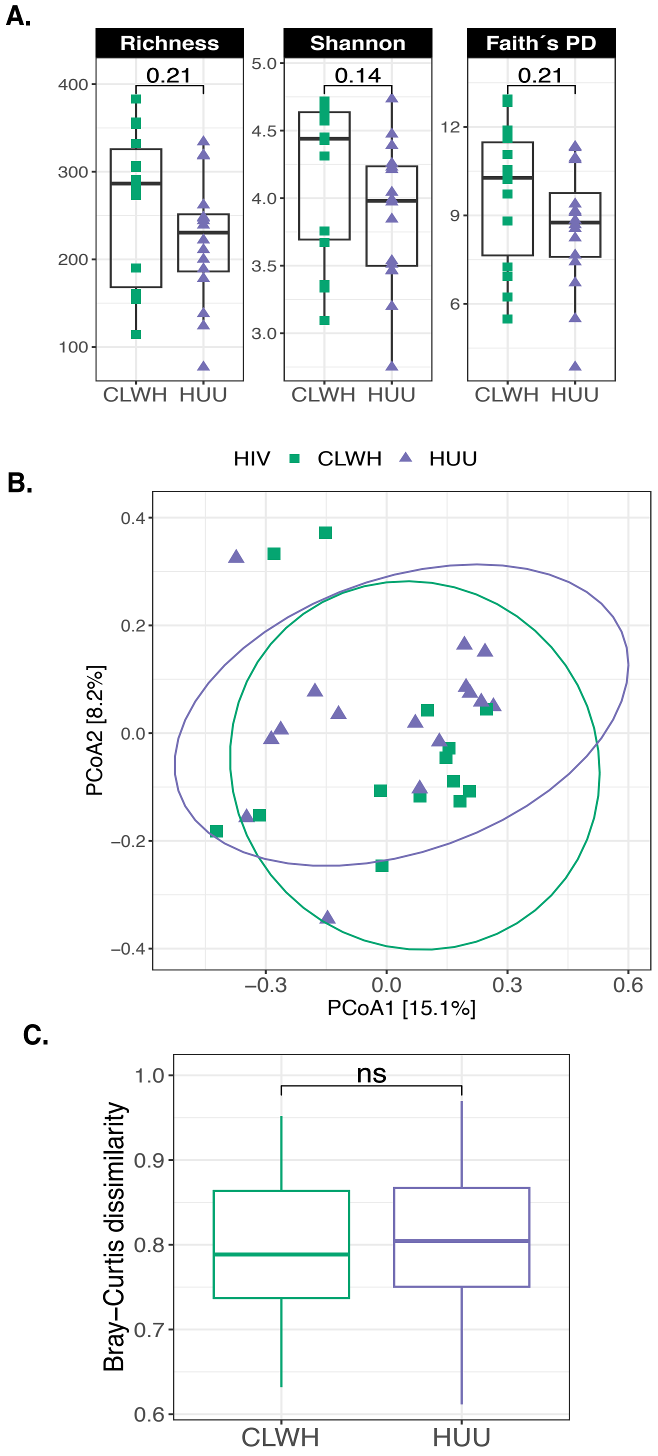

Supplementary Figure 2. A. Alpha diversity was higher in CLWH for all three indices (richness, shannon and phylogenetic distance), although no differences were found when comparing CLWH to HUU using the Wilcoxon Rank Sum Test. B. No clear evidence of clustering by HIV status (R-squared= 0.034, PERMANOVA p=0.46) was found. C. The interindividual Bray-Curtis dissimilarity index was similar between CLWH and HUU.

Alpha and beta diversity were performed after rarefaction (59,126 sequences/sample). For beta diversity, the Bray-Curtis dissimilarity index was estimated. Graphs were done in R version 4.3.0 using ggplot2 version 3.4.2.

Abbreviations: CLWH: children living with HIV, HUU: HIV-unexposed and uninfected children, PCoA: principal coordinate analysis, PD: phylogenetic distance, PERMANOVA: Permutational Multivariate Analysis of Variance

Supplementary Table 6. No association between HIV status and alpha diversity metrics

	**Univariate analysis**		**Adjusted for sex, age, and BMI**		**Adjusted for sex, age, BMI, and recent antibiotic use**	
	β coefficient (95% CI)	p value	β coefficient (95% CI)	p value	β coefficient (95% CI)	p value
Richness	39.48 (-20.38- 99.35)	0.188	56.30 (-6.29-118.90)	0.075	55.76 (-13.53-125.07)	0.109
Shannon	0.28 (-0.12-0.70)	0.169	0.43 (-0.002-0.86)	0.051	0.43 (-0.04-0.91)	0.075
Faith PD	1.11 (-0.52-2.79)	0.187	1.45 (-0.36-3.31)	0.112	1.53 (-0.50-3.57)	0.097

Linear regression analysis between the outcome variable (alpha diversity metrics) and HIV status (reference: CLWH). The univariate model was adjusted for sex, age and BMI/A (model 1), and sex, age, BMI/A and recent antibiotic use (antibiotics used in the 3 previous months) (model 2).

Abbreviations: CI: %: percentage, BMI: body mass index, CI: confidence interval

Supplementary Figure 3. Relative abundance (%) at phylum level stratified by HIV status and sex

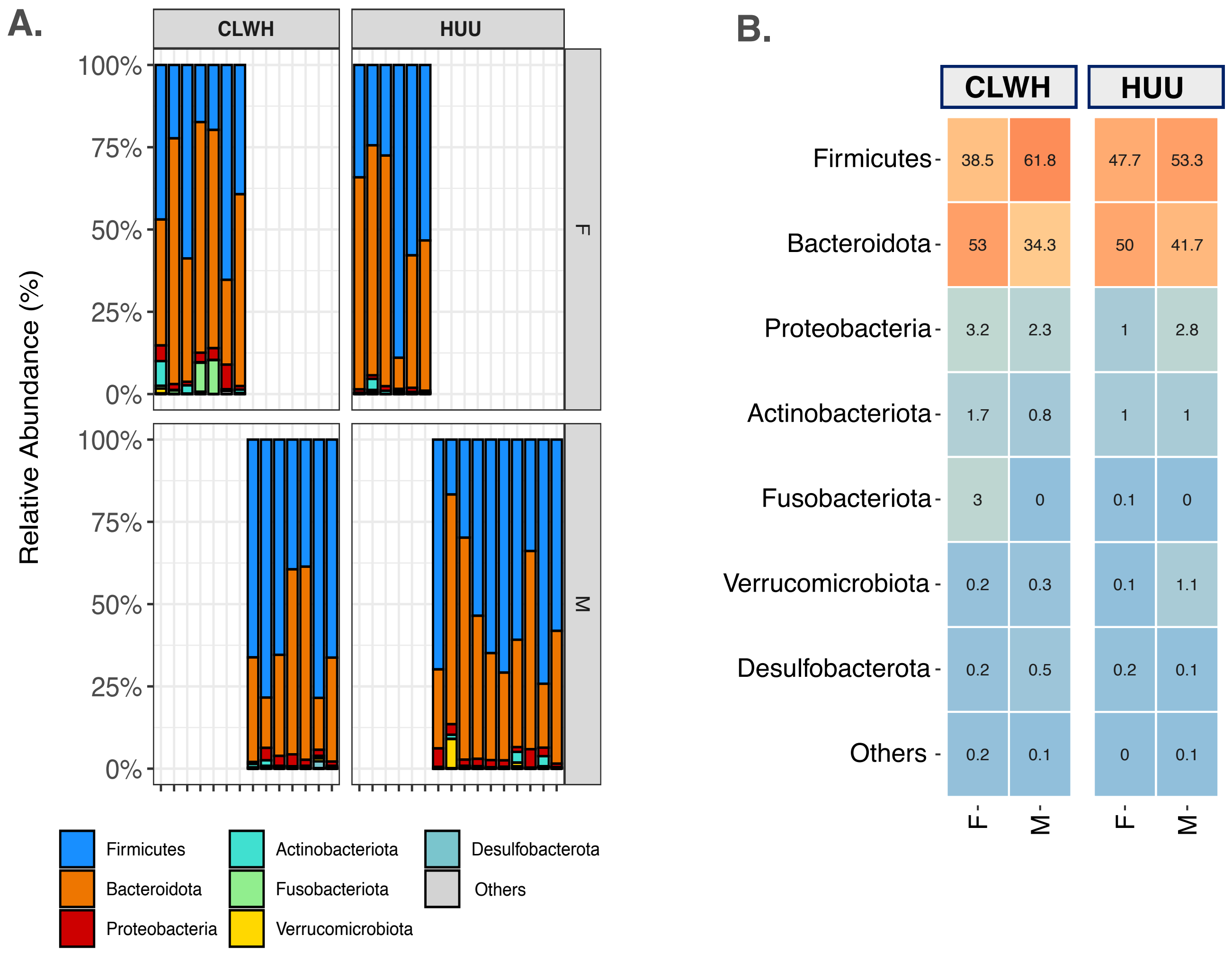

Supplementary Figure 3. The relative abundance (%) at phylum level is shown stratified by HIV status and faceted by sex assigned at birth. A. Each barplot represents a child. Children were also ordered by age, from youngest to oldest. B. Heatmap showing the overall relative abundance (shown) in each group: CLWH F, CLWH M, HUU F, and HUU M. Firmicutes and Bacteroidota are predominant, accounting for 94.95% of all phyla present. Proteobacteria, Actinobacteria, Fusobacteria, Verrucomicrobiota, Desulfobacterota account for 4.69%. Others refers to the remaining 5 phyla that collectively accounted for less than 0.1% relative abundance. Barplots and heatmap were created using ggplot2 version 3.4.2 and ampvis2 version 2.8, respectively in R version 4.3.0.

Abbreviations:
CLWH: children living with HIV, F: female, HUU: HIV-unexposed and -uninfected children, M: male

Supplementary Figure 4. Heatmap showing the relative abundance of the top 40 genera in each child

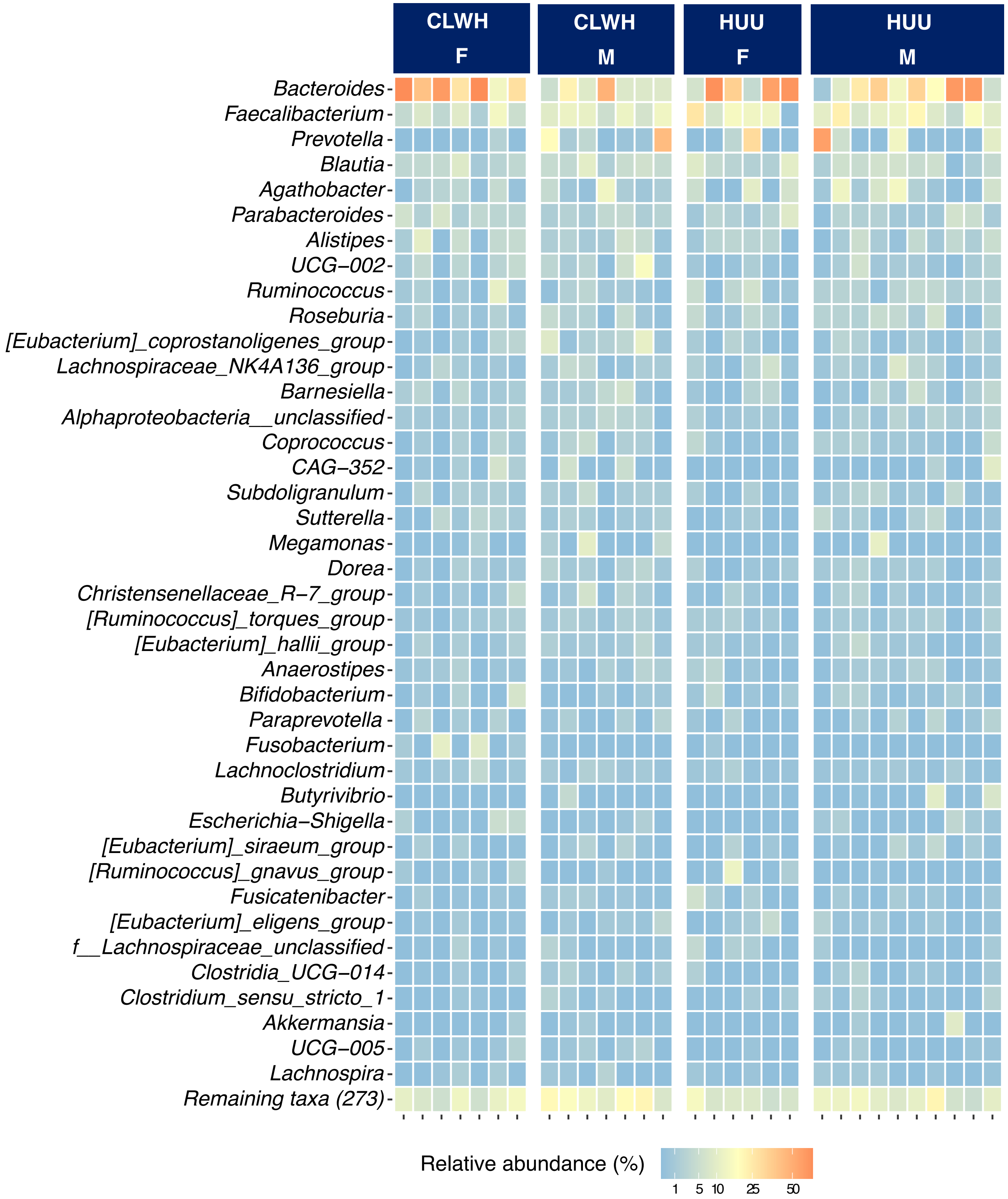

Supplementary Figure 4. The relative abundance (%) of the top 40 genera is shown stratified by HIV status and faceted by sex assigned at birth. The top 40 genera account for 89.44% of all genera present. Heatmap was created using ampvis2 version 2.8 in R version 4.3.0.

Abbreviations: CLWH: children living with HIV, F: female, HUU: HIV-unexposed and -uninfected children, M: male

Supplementary Table 6. History of previous antibiotic usage in CLWH and HUU

	CLWH		HUU	
**Exposure to Antibiotics**				
Recent exposure to Abx	**YES**	**NO**	**YES**	**NO**
Number of children with recent exposure to Abx	4	11	8	8
Time between last Abx and sample collection (days)	9 (2.5-14.5)	148 (115-365)	17 (2-19.5)	97.5 (92-240)
**Antibiotic class**				
β-lactams	4 (80)	-	3 (33.3)	-
Cephalosporins	1 (20)	-	2 (22.2)	-
Sulfonamides	0	-	2 (22.2)	-
Lincosamides	0	-	2 (22.2)	-
**Reason for prescribing antibiotics**				
Infection of the upper respiratory tract<br>Infection of the gastrointestinal tract<br>Others	4 (100)<br>0 (0)<br>0 (0)	-	5 (50)<br>3 (30)<br>2 (20)	-
Total number of antibiotic courses since birth	2 (1 – 4)	2 (1 – 4)	5 (3 – 5)	3 (2 – 5)

Data are expressed as number (percentage) or median (interquartile range) as appropriate.

Abbreviations

β : beta, CLWH: children living with HIV, HUU: HIV-uninfected and -unexposed.

Supplementary Table 7. Stool consistency (Bristol Stool Chart Form)

	CLWH		HUU	
Type 1	0 (0)	1 (9.1)	1 (12.5)	0 (0)
Type 2	0 (0)	3 (27.3)	2 (25)	2 (25)
Type 3	1 (25)	1 (9.1)	1 (12.5)	3 (37.5)
Type 4	2 (50)	6 (54.5)	3 (37.5)	1 (12.5)
Type 5	1 (25)	0 (0)	0 (0)	1 (12.5)
Type 6	0 (0)	0 (0)	1 (12.5)	1 (12.5)
Type 7	0 (0)	0 (0)	0 (0)	0 (0)

Data are expressed as number (percentage). Recordings were made by the same untrained observer, before processing the fecal sample.

Abbreviations
CLWH: children living with HIV, HUU: HIV-uninfected and -unexposed.

Supplementary Table 8. Impact of variables known to influence the gut microbiota as assessed by permutational multivariate analysis of variance

Variables	Categories	R-squared	Pseudo-F	PERMANOVA
HIV status	CLWH, HUU	0.034	0.9864	0.4609
Sex	Female, Male	**0.052**	1.5601	**0.024**
Age		0.042	1.245	0.118
ART	HUU, PI, INSTI, NNRTI	0.090	0.866	0.870
BMI/A		0.031	0.896	0.642
Previous antibiotic usage	Yes, No	0.034	1.005	0.422
Place of residence	CDMX, EDOMEX, Other	0.079	1.164	0.141
Immune status (CD4)		0.033	0.978	0.474
Mode of delivery	Vaginal, C-section	0.033	0.9676	0.493
Maternal feeding	Breastfed, Formula, Mixed	0.079	1.173	0.130
Siblings	Yes, No	0.029	0.855	0.749
Siblings (#)		0.026	0.763	0.897
Stool consistency	Type 1 to Type 7	0.153	0.873	0.910

Permutations were set to 10,000.
Abbreviations
#: number, ART: Antiretroviral therapy, BMI/A: body mass index for age (z score), CLWH: Children living with HIV, CDMX: Ciudad de México, EDOMEX: Estado de México, F: female, HUU: HIV-unexposed and -uninfected children, M: male, PERMANOVA: permutational multivariate analysis of variance

Supplementary Table 9. Associations between sex assigned at birth and Firmicutes, *Bacteroides*, and microbial richness are lost in adjusted regression analysis

Outcome variable	Univariate analysis		Adjusted for HIV status, age, and BMI		Adjusted for HIV status, age, BMI, and recent antibiotic use	
	β coefficient (95% CI)	p value	β coefficient (95% CI)	p value	β coefficient (95% CI)	p value
Firmicutes	0.14 (-0.009-0.28)	**0.064**	0.10 (-0-07-0.27)	0.232	0-09 (-0.10-0.28)	0.341
Bacteroides	-0.19 (-0.35 to -0.03)	**0.019**	-0.19 (-0.35 to -0.03)	0.139	-0.11 (-0.30-0.08)	0.254
Richness	61.20 (6.17-116.24)	**0.037**	57.50 (-6.16-121.17)	0.074	56.97 (-13.42-127.36)	0.107

Linear regression analysis between outcome variables and sex assigned at birth (reference males). The univariate model was adjusted for sex, age and BMI/A (model 1), and sex, age, BMI/A and recent antibiotic use (antibiotics used in the 3 previous months) (model 2)

Abbreviations: %: percentage, BMI: body mass index, CI: confidence interval, HIV: human immunodeficiency virus
